# Safe Stimulant Prescribing in Postural Orthostatic Tachycardia Syndrome (POTS): A Case Highlighting Multidisciplinary and System-Level Safeguards

**DOI:** 10.1192/bjo.2026.11883

**Published:** 2026-06-30

**Authors:** Gayathri Rangith

**Affiliations:** Cygnet Health Care, Oldbury, United Kingdom

## Abstract

**Aims:**

Prescribing stimulant medication for Attention-Deficit/Hyperactivity Disorder in patients with Postural Orthostatic Tachycardia Syndrome presents a recognised clinical dilemma due to the potential for stimulants to exacerbate tachycardia and autonomic instability. There is limited practical guidance on how such prescribing can be undertaken safely within psychiatric inpatient settings. This case aims to demonstrate how structured multidisciplinary collaboration, robust monitoring protocols, and clear system ownership can enable safe initiation of stimulant treatment in a patient with POTS, while highlighting vulnerabilities in protocol implementation within complex care systems.

**Methods:**

A woman in her early twenties was admitted to an acute psychiatric ward with complex psychiatric and medical comorbidities, including POTS (stable on Ivabradine), Epilepsy, and Schizoaffective disorder. Following comprehensive assessment, she was diagnosed with ADHD using the DIVA-5 diagnostic interview.

Given the cardiovascular risks associated with stimulant medication, extensive liaison was undertaken with multiple consultant cardiologists at a tertiary NHS trust. Cardiology confirmed stable POTS, a normal recent ECG, and no evidence of structural heart disease, advising that Lisdexamfetamine 20 mg once daily could be initiated with appropriate precautions.

A detailed monitoring protocol was developed and disseminated, including baseline and thrice-daily lying, sitting, and standing heart rate and blood pressure measurements, NEWS2 scoring, predefined red-flag thresholds, and a clear escalation plan. The patient received structured education regarding symptom reporting, and informed written consent was obtained. The plan was shared with nursing staff and on-call medical teams to support safe implementation.

**Results:**

Lisdexamfetamine was initiated without cardiovascular complications. During the initial two-week intensive monitoring period, no tachycardia, blood pressure instability, or worsening of POTS symptoms were observed. The patient reported significant subjective improvement in ADHD symptoms, corroborated by staff observations. Ongoing weekly monitoring continued following dose changes.

A systems-based challenge was identified when elements of the monitoring protocol were not implemented consistently during a period of medical handover, despite prior written communication. This highlighted a critical gap between protocol dissemination and execution, underscoring the importance of clear ownership and active follow-up when implementing high-risk prescribing plans.

**Conclusion:**

This case demonstrates that stimulant medication can be safely initiated in selected patients with POTS when supported by specialist cardiology input, structured monitoring, and robust multidisciplinary collaboration. However, safe prescribing extends beyond clinical decision-making alone; system-level safeguards, clear accountability, and effective handover processes are essential to mitigate risk. Patient education and proactive MDT engagement are pivotal in ensuring both safety and therapeutic benefit in complex psychiatric prescribing.

